# CRISPR/Cas9-Based therapeutics as a promising strategy for management of Alzheimer’s disease: progress and prospects

**DOI:** 10.3389/fncel.2025.1578138

**Published:** 2025-04-07

**Authors:** Mohamad Sultan Khan, Nousheen Qureshi, Rehan Khan, Young-Ok Son, Tariq Maqbool

**Affiliations:** ^1^Laboratory of Nanotherapeutics and Regenerative Medicine, Department of Nanotechnology, University of Kashmir, Srinagar, India; ^2^Department of Higher Education, Government of Jammu and Kashmir, Srinagar, India; ^3^Chemical Biology Unit, Institute of Nano Science and Technology, Knowledge City, Mohali, Punjab, India; ^4^Department of Animal Biotechnology, Faculty of Biotechnology, College of Applied Life Sciences and Interdisciplinary Graduate Program in Advanced Convergence Technology and Science, Jeju National University, Jeju, Republic of Korea

**Keywords:** CNS, Alzheimer’s disease, gene editing (CRISPR/Cas9), therapeutics, nanocarriers

## Abstract

CRISPR/Cas9 technology has revolutionized genetic and biomedical research in recent years. It enables editing and modulation of gene function with an unparalleled precision and effectiveness. Among the various applications and prospects of this technology, the opportunities it offers in unraveling the molecular underpinnings of a myriad of central nervous system diseases, including neurodegenerative disorders, psychiatric conditions, and developmental abnormalities, are unprecedented. In this review, we highlight the applications of CRISPR/Cas9-based therapeutics as a promising strategy for management of Alzheimer’s disease and transformative impact of this technology on AD research. Further, we emphasize the role of CRISPR/Cas9 in generating accurate AD models for identification of novel therapeutic targets, besides the role of CRISPR-based therapies aimed at correcting AD-associated mutations and modulating the neurodegenerative processes. Furthermore, various delivery systems are reviewed and potential of the non-viral nanotechnology-based carriers for overcoming the critical limitations of effective delivery systems for CRISPR/Cas9 is discussed. Overall, this review highlights the promise and prospects of CRISPR/Cas9 technology for unraveling the intricate molecular processes underlying the development of AD, discusses its limitations, ethical concerns and several challenges including efficient delivery across the BBB, ensuring specificity, avoiding off-target effects. This article can be helpful in better understanding the applications of CRISPR/Cas9 based therapeutic approaches and the way forward utilizing enormous potential of this technology in targeted, gene-specific treatments that could change the trajectory of this debilitating and incurable illness.

## 1 Introduction

The central nervous system (CNS) disorders, including neurodegenerative diseases, psychiatric conditions, and developmental disorders, are considered the major global health care challenges (DiNunzio and Williams, 2008; Naz and Siddique, 2020; Zahra et al., 2020; Ayeni et al., 2022). These diseases often have multifactorial etiologies involving intricate genetic and molecular mechanisms (Migliore and Coppedè, 2009). Understanding the pathophysiology of these conditions is critical for developing effective therapeutic interventions (Gribkoff and Kaczmarek, 2017; Reddy and Abeygunaratne, 2022; Kashyap et al., 2023). However, research on CNS diseases is challenging because of the presence of the blood–brain barrier (BBB) and complexity of neural networks (Sweeney et al., 2019; Alajangi et al., 2022). Although advancements in molecular biology and genetics have expanded our understanding of CNS diseases, identifying therapeutic targets for these disorders has been rather slow. In this context, genome-editing technologies, particularly CRISPR/Cas9, have emerged as pivotal tools for elucidating the molecular mechanisms underlying CNS pathologies, offering promising avenues for the development of novel and targeted therapeutic strategies. This system has revolutionized our ability to edit genes precisely, offering a powerful tool to decipher targets for CNS diseases (Powell et al., 2017; Guan et al., 2022; Jiang et al., 2024). Herein, we provide a comprehensive overview of the CRISPR/Cas9 technology and the recent progress made in its application in AD research.

## 2 The CRISPR/Cas9 system and gene editing

**FIGURE 1:**
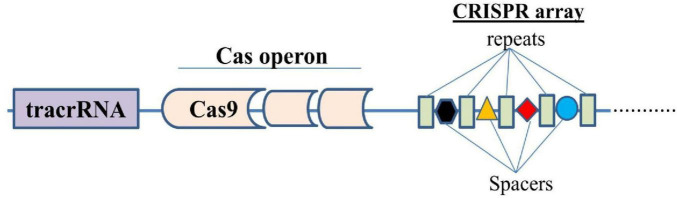
Simplified structure of a typical CRISPR/Cas system.

The CRISPR/Cas9 system was discovered as a prokaryotic adaptive immune system, specifically in bacteria and archaea (Barrangou et al., 2007). CRISPR, an acronym for “Clustered regularly interspaced short palindromic repeats,” refers to short and repetitive DNA sequences found in the genomes of bacteria and some archaea (Barrangou, 2015). The discovery of CRISPR-Cas9 system and its transformation into a potent DNA editing tool has unfolded great potential therapeutic opportunities to address the genetic root causing diverse diseases including CNS disorders such as Alzheimer’s, and Parkinson’s diseases (Chen et al., 2019; De Plano et al., 2022). This system involves the endonuclease Cas9 bound to a short RNA called single guide RNA (sgRNA) that targets a specific genomic region of interest, by complementary base pairing and cleaves the DNA, resulting in a double-strand DNA break (DSB) at the binding site. This DSB can be exploited for gene knockout, correction, deletion, or addition, which can be repaired through different cellular mechanisms of DNA repair as the homology-directed repair (HDR) using a DNA template with homologous arms and the error-prone end-joining repair pathways such as non-homologous end joining (NHEJ) that introduces indels and frameshifts (Chen et al., 2019). A typical CRISPR locus in a type II CRISPR/Cas system comprises an array of repetitive sequences (*repeats*, blue colored structures) interspaced by short stretches of nonrepetitive sequences (*spacers*, colored different structures), and a Cas operon, a set of CRISPR-associated (*cas*) genes (Figure 1). Preceding the *cas* operon is the *trans*-activating CRISPR RNA (*tracrRNA*) gene, which encodes a unique noncoding RNA with homology to the repeat sequences (Jiang and Doudna, 2017). A special enzyme known as Cas9 (CRISPR-associated protein 9) uses CRISPR sequences as a guide to recognize and unwind specific strands of DNA that are complementary to these (CRISPR) sequences. Hence, CRISPR in conjunction with Cas9 forms the CRISPR/Cas9 system, which is a defense mechanism adapted for gene editing in bacteria and archaea (Reeks et al., 2013; Sorek et al., 2013; Barrangou and Marraffini, 2014; Barrangou, 2015). Cas9 consists of six domains, namely REC I, REC II, bridge helix, protospacer adjacent motif (PAM)-interacting domain, HNH domain, and RuvC. The REC I domain facilitates the binding of the guide RNA (gRNA) to the target sequence, the PAM domain helps in starting the interaction, whereas the arginine rich bridge helix is essential for starting cleavage activity upon engagement of target DNA. A chimeric single guide RNA (sgRNA) can be created by fusing the CRISPR RNA (crRNA)–tracrRNA duplex (Jinek et al., 2012; Nishimasu et al., 2014).

### 2.1 Mechanism of gene editing by CRISPR/Cas9

**FIGURE 2:**
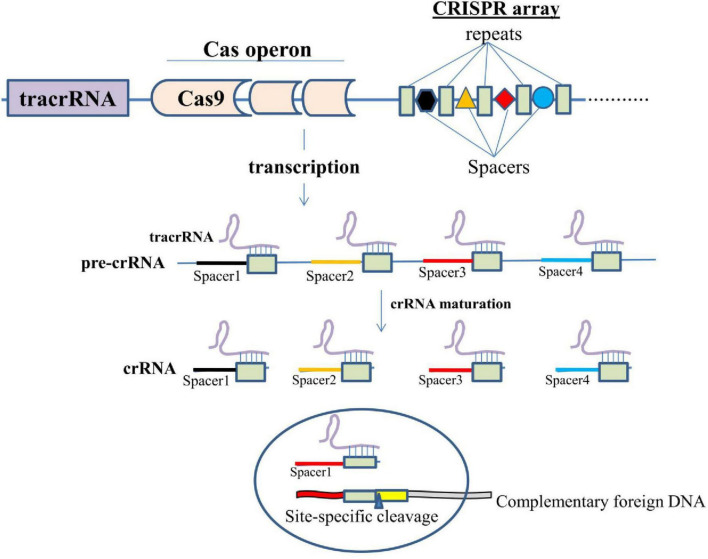
Diagrammatic representation of the mechanism of action of CRISPR/Cas9.

To understand the mechanism of gene editing using the CRISPR/Cas9 technology, it is essential to understand the basic mechanism of action of this system. The acquisition machinery (Cas enzymes) incorporates a new spacer from the invasive genetic material into the CRISPR. The genetic elements from phage or plasmids, or short fragments of foreign DNA are integrated into the CRISPR repeat-spacer array within the host chromosome as new spacers. Following integration, the new spacer cotranscribes with every other spacer to create a lengthy precursor CRISPR RNA (pre-crRNA) that comprises spacers and repeats. After being independently transcribed, the tracrRNA anneals to the pre-crRNA repeats for maturation into crRNA via RNase III cleavage. The guide sequence is 20-nucleotide long after additional trimming of the 5′-end of the crRNA (gray arrowheads) by unidentified nucleases (Figure 2). Cas9 endonuclease is bound by the mature crRNA–tracrRNA complex during interference, and it is further directed to cleave foreign DNA that has a complementary 20-nucleotide crRNA sequence before the PAM sequence. Three essential elements make up the widely used type II CRISPR/Cas9 system: tracrRNA, crRNA, and the endonuclease Cas9 (Jinek et al., 2012).

CRISPR/Cas9 system has been successfully used to make site-specific cuts in target DNA, introduce mutations, or correct genetic errors as discovered by Doudna and Charpentier in 2012 (Jinek et al., 2012). This technique facilitates precise gene editing with an unparalleled degree of precision and effectiveness (Jinek et al., 2012; Hermann, 2020; Hermann, 2023). The gene-editing capability has made CRISPR/Cas9 a significant tool for studying CNS-related disorders by allowing the manipulation or alterations of disease-associated genes, creating models to study their function, and screening for potential therapeutic targets. Consequently, over the last decade, our understanding of the CRISPR/Cas9 biology and its applications has made this technology a fundamental tool gene editing tool in various organisms (Wright et al., 2016). The versatility of this technology has rapidly accelerated the pace of CNS research, enabling the modeling of genetic mutations implicated in CNS-related disorders and offering new avenues for therapeutic interventions.

### 2.2 Various delivery system used for CRISPR-Cas9

Despite the great promise of the CRISPR-Cas9 system for site-specific gene editing (Adli, 2018), one of the challenges is developing biocompatible and safe method to deliver CRISPR-Cas9 RNA *in vivo* gene therapy. Another challenge is the risk of nuclease degradation and rapid CRISPR-Cas9 system clearance by macrophages, the large size of Cas9, the high anionic charge density, and hydrophilic nature of the sgRNA, which imposes a significant barrier to their efficient nuclear delivery and overall efficiency of gene transfection (Glass et al., 2018). CRISPR-Cas9 system can be delivered by following approaches to the cells: (i) plasmid encoding for Cas9 and sgRNA (Nakamura et al., 2019), (ii) mRNA encoding for Cas9 and sgRNA (Miller et al., 2017), and (iii) Cas9 ribonucleoprotein (RNP) and sgRNA (Mout et al., 2017). Notably, delivering a plasmid DNA encoding for the Cas9 protein and its sgRNA is highly stable and more cost-effective than the RNP form or mRNA. However, it must overcome the challenge of crossing the cellular and nuclear membranes besides avoiding the possibility of random integration into the genome and activation of unwanted immune responses due to the long target-editing time of Cas9 mRNA (Zuckermann et al., 2015). Moreover, delivering Cas9 protein directly with the sgRNA is the most straightforward and typically applied strategy but due to the large size of Cas9 protein this approach is challenging *in vivo* due to the risk of immunological response (Liang et al., 2015). A second approach provides an interesting alternative, as the mRNA encoding for Cas9 results in rapid gene editing and avoids the challenge of nuclear internalization and transcription process. Moreover, the risk of off target effects is decreased and integration into the genome is removed by the transient protein expression. Although, mRNA is less stable than the DNA or RNP forms, its encapsulation into nanoparticle-based delivery systems can protect it from RNAse degradation *in vitro* and *in vivo* (Chen et al., 2019).

Various delivery systems for CRISPR/Cas9 system are based on either viral or non-viral vectors, each with distinct advantages and limitations (Xu et al., 2019; Barman et al., 2020; Mengstie, 2022; Taha et al., 2022; Taghdiri and Mussolino, 2024).

Viral delivery methods for CRISPR/Cas9 include viral vectors such as lentivirus, adenovirus, and adeno-associated virus (AAV). These systems are highly efficient at delivering the CRISPR components to both dividing and non-dividing cells and can achieve stable integration of the CRISPR/Cas9 components into the host genome (Mengstie, 2022; Li et al., 2023; Taghdiri and Mussolino, 2024). However, viral delivery methods come with certain drawbacks. For example, immune responses to the viral vectors, the potential for insertional mutagenesis, and safety concerns related to their use in clinical applications limit their widespread adoption. Moreover, viral vectors often have a limited capacity for carrying large payloads, which can hinder the delivery of complex or large CRISPR constructs (Gaj et al., 2016; Sun et al., 2019; Ghosh et al., 2020; Mengstie, 2022; Taghdiri and Mussolino, 2024).

Non-viral delivery systems for CRISPR/Cas9 includes a variety of approaches such as electroporation, microinjection, and nano-based delivery systems such lipid nanoparticles (LNPs), Gold based nanoparticle, peptide-based nanoparticles, DNA nanoclews, polymeric nanocomplexes, etc. (Park et al., 2019; Wang et al., 2020; Shaikhutdinov et al., 2024). Non-viral delivery systems for CRISPR/Cas9 gene editing have gained significant attention due to their lower risk of immune activation and insertional mutagenesis, making them safer alternatives to viral methods. Nano-based delivery systems enable efficient encapsulation of the different CRISPR/Cas9 cargos and *in vivo* therapeutic efficacy of CRISPR/Cas9 with controlled size, shape and surface charge have been developed (Duan et al., 2021b). Non-viral delivery systems for CRISPR/Cas9 gene editing have gained significant attention due to their lower risk of immune activation and insertional mutagenesis, making them safer alternatives to viral methods. Electroporation involves applying an electrical field to facilitate the uptake of CRISPR/Cas9 components, is another widely used technique, though it often results in transient expression and can be detrimental to cell viability, particularly at high voltages (Rathbone et al., 2022). Microinjection directly injects CRISPR/Cas9 constructs into the nucleus, providing a high efficiency of genome editing in certain cell types but at the cost of being labor-intensive and technically challenging (Liu et al., 2017). Various nanoparticles including gold nanoparticles offer additional innovative solutions providing a versatile, scalable method of delivery due to their stability and ease of production. DNA nanoclews are a type of nanoparticles constructed by assembling short strands of DNA into stable nanostructures, which can encapsulate or carry CRISPR components into cells (Rawal et al., 2024). Furthermore, polymeric nanocomplexes, which involve CRISPR components being complexed with polymers, can improve the stability and cellular uptake of the genetic material, although they still face challenges related to efficiency and cytotoxicity (Shareena et al., 2025). Lipid nanoparticles (LNP) have shown great promise in enhancing the stability of the CRISPR-Cas9 machinery as well as improving its bioavailability *in vivo* (Zhang et al., 2017). LNPs consist of lipid-based particles that encapsulate the CRISPR components, facilitating their entry into cells via endocytosis (Barman et al., 2020). More recently, a promising new Peptide-Based Nanoparticles for delivering CRISPR-Cas9 system, using a new generation of cell penetrating peptides called “ADGN peptides”, able to form stable nanoparticles with long mRNA and CRISPR components was reported (Gonzalez et al., 2024). These ADGN peptides possess great potential as a cellular carrier of CRISPR-Cas9 system for gene editing *in vitro* and *in vivo* in mice models. It was shown that ADGN nanoparticles were able to overcome delivery barrier by bringing a real protection to RNA and delivering a functional and efficient CRISPR Cas9 system in tumoral cells implanted into mouse lung. Furthermore, it was demonstrated that compared to LNPs, these peptide-based nanoparticles (PBN) have ability for systemic extrahepatic delivery of therapeutic nucleotides, chemical diversity and functional potential (Wickline et al., 2023). They provide more choices for flexible structural designs to deliver different nucleic acid types as well as to develop targeting strategies and consequently are gaining more and more interest and appear as a promising alternative (Guan et al., 2018).

A comprehensive analysis of the important viral and non-viral delivery systems, including their respective advantages and limitations, is provided in Table 1 for further details.

**TABLE 1:** Different CRISPR/Cas9 delivery systems in Alzheimer’s disease.

Vectors used	Advantages	Disadvantages	References
AAV	Minimally immunogenic; high delivery ability; cannot integrate into the human genome	Incorporateslower-sized genomes (up to 4.7 kb); usually requires coinjection of other viruses as carriers	Grimm and Kay, 2003; Recchia et al., 2004; Gaj et al., 2016
Lentivirus	Can incorporate long DNA inserts (8–10 kb); can target genes in both sAD and fAD, including APP, APOE4, and caspase-6	Difficult to purify in large quantities; can provoke immune response; can integrate into the human genome	Recchia et al., 2004; Dissen et al., 2012; Offen et al., 2018a,b; Sun et al., 2019
Injections of nanocomplexes (as intrathecal and ICV)	Less immunogenic; easy formation; cost effective; Nonsignificant off-target mutation rate	Less efficient; require multiple injections; cannot cross BBB; removed from the blood by RES.	Park et al., 2019
Polymeric nanocomplexes of PMT and RVG	Improved conveyance ability; can cross BBB	Diminished transfection efficiency due to the presence of PEG	Dissen et al., 2012
DNA nanoclews	Can be used in conveying the Cas9–sgRNA complex	Complicated and time-consuming; induce immunogenic reactions	Sun et al., 2015
Lipid and polymeric NPs	Not much details available	Not much details available	Kulkarni et al., 2018; Aghamiri et al., 2020
Gold NPs	High tolerability and low toxicity	Not much details available	Wang et al., 2020
Micro vesicles	Cost effective; reusable	Low efficiency	Anzalone et al., 2019

PMT, poly mannitol-copolyethylenimine; RVG, rabies infection glycoprotein; BBB, blood brain barrier; RES, reticuloendothelial system; ICV, Intracerebroventricular injections; PEG, polyethylene glycol; NPs, nanoparticles.

## 3 Central nervous system disorders

**FIGURE 3:**
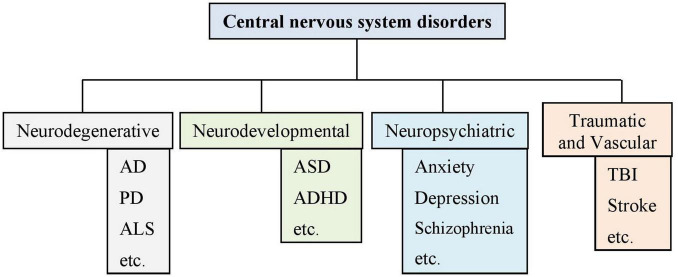
Schematic representation of several important central nervous system-related disorders. AD: Alzheimer’s disease; PD: Parkinson’s disease; ALS: Amyotrophic lateral sclerosis; ASD: Autism spectrum disorder; ADHD: Attention-deficit/hyperactivity disorder; TBI: Traumatic brain injury.

Central nervous system (CNS) disorders encompass a wide range of conditions, including neurodegenerative, neurodevelopmental, and neuropsychiatric disorders as represented in a schematic diagram in Figure 3. Neurodegenerative disorders, such as Alzheimer’s disease (AD) and Parkinson’s disease (PD), are characterized by progressive neuronal dysfunction and death in various brain regions specific to the disease condition. AD, the most common form of dementia, is linked to amyloid plaque and tau tangles, leading to cognitive decline (Thal et al., 2014; Mumtaz et al., 2022; Ferrer, 2023). PD involves the degeneration of dopaminergic neurons, causing motor impairments and other symptoms (Guatteo et al., 2022). Amyotrophic lateral sclerosis (ALS) results in motor neuron death and paralysis, often due to genetic and environmental factors (Kiernan et al., 2011; Grad et al., 2017). Neurodevelopmental disorders, such as Autism spectrum disorder (ASD) and Attention-Deficit/Hyperactivity Disorder (ADHD), emerge during brain development and are associated with disruptions in neural connectivity and neurotransmitter signaling (Lord et al., 2018; Moschetti et al., 2024; Turiaco et al., 2024). Neuropsychiatric disorders like schizophrenia and depression impact brain function, affecting mood, cognition, and behavior, and are influenced by genetic, environmental, and neurochemical factors (Kirov, 2015; Kasem et al., 2018; McCutcheon et al., 2020; Thapar et al., 2024). Traumatic brain injury (TBI) and stroke, resulting from physical trauma or vascular injury, lead to cognitive and motor dysfunction (Nolan, 2005; Blennow et al., 2016; Dadas et al., 2018; Murphy and Werring, 2020).

This review focuses on AD, applications of CRISPR/Cas9-based therapeutics as a promising strategy for management of Alzheimer’s disease and transformative impact of this technology on AD research in the following sections.

## 4 Alzheimer’s disease

AD is a neurodegenerative disorder characterized by progressive neuronal loss due to diminished regenerative capacity of neurons, leading to brain atrophy, dementia, and ultimately death of an individual (Mumtaz et al., 2022). Nearly 35 million people are estimated to be suffering from AD worldwide and this number is predicted to double by 2030. This disease demands proper and long-term medical care, which accounted for an estimated USD195 billion in 2019 and this cost is expected to increase to USD1 trillion by 2050 (Haque and Levey, 2019; Mumtaz et al., 2022). Although the specific cause of this disease is not known, the patients exhibit the presence of Aβ as plaques and hyperphosphorylated tau proteins, as NFTs in multiple regions of the brain. Although accumulation of these proteins is a major pathological hallmark of AD, the failure to cure this dreadful disease has drawn special attention of researchers to search for more suitable biological markers.

### 4.1 Proteins and enzymes involved in the progression of AD

**FIGURE 4:**
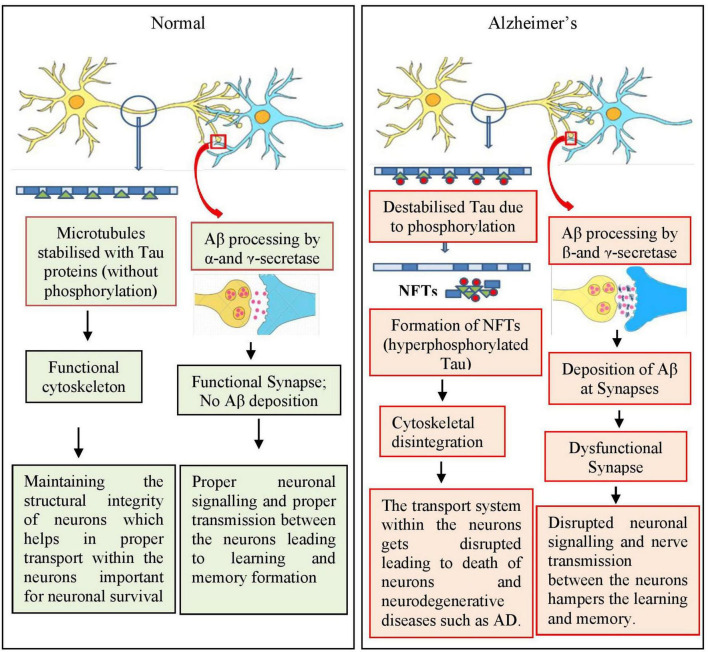
Simplified mechanism involved in amyloid beta deposition and tau phosphorylation during the progression of Alzheimer’s disease (AD). During normal conditions, APP is processed involving α- and γ-secretase enzymes and the cytoskeleton of neurons, that is microtubules, are stabilized by Tau proteins leading to functional and healthy neurons and neuronal network. In AD condition, APP is processed by β- and γ-secretase enzymes leading to the formation of amyloid plaques between the synapses, blocking the neuronal network. Tau proteins are also hyperphosphorylated leading to destabilized microtubules contributing toward nonfunctional neuronal network. = Tau; = phosphorylation; = microtubules.

Various proteins and enzymes are involved in AD progression (as reviewed by Mumtaz et al., 2022). The most important protein, Aβ, is derived from amyloid-beta precursor protein (APP), which is encoded by the *APP* gene. APP is a transmembrane protein that is predominantly expressed in neurons, astrocytes, and vascular endothelial cells (Miura et al., 2020; Zhao et al., 2020; Zhou F. et al., 2020). The expression of *APP* increases during neuronal differentiation and synaptic junction formation, and starts decreasing once mature connections are established, predicting its possible role in aging and neuronal development (Chen et al., 2017). The generation of soluble (non-pathogenic) and insoluble Aβ fragments (pathogenic) generated in normal and diseased conditions, respectively, remains unknown. Under normal conditions, APP undergoes non-pathogenic processing with the help of two enzymes α-and γ-secretase, whereas in diseased/AD conditions, a distinct group of enzymes, namely β-and γ-secretase, are involved (Nesterova et al., 2019). Additionally, any mutation in APP gene can cause AD, especially early-onset-familial AD (Hooli and Tanzi, 2016; Figure 4).

Another enzyme, beta-site APP-cleaving enzyme (BACE) has been reported to be involved in the proteolytic cleavage of APP for the production of neuropathogenic Aβ peptides (Mowrer and Wolfe, 2008). BACE cleaves APP, generating a C99 membrane-bound C-terminal fragment, which is further processed by γ-secretase, leading to the formation of Aβ peptides (Bolduc et al., 2016). BACE is highly expressed in various parts of the brain of patients with AD, especially in the cortex and cerebrospinal fluid (Hampel et al., 2020).

Presenilin 1 and 2 (PSEN 1 and 2) are the components of γ-secretase. Mutations in presenilin genes (*PSEN1* and *PSEN2*) causes structural and functional alterations in γ-secretase and hence increase the production of Aβ fragments (Xia et al., 2015; Zhang et al., 2015; Giri et al., 2016; Miki et al., 2019; Escamilla-Ayala et al., 2020; Andrade-Guerrero et al., 2023).

Tau is a microtubule-associated protein involved in the stabilization of microtubules present within the neurons. It regulates neuronal cytoskeleton and also helps in the axonal transport (Zhou et al., 2018; Zhou F. et al., 2020; Zhou Y. et al., 2020). Any alterations in the structural conformation, which include hyper phosphorylation, can impact its binding with microtubule leading to its dissociation from microtubules and toxic aggregation in the form of NFTs (Figure 2). These NFTs, upon aggregation, attain the shape of paired helical filaments (PHFs), which are one among the major hallmarks of AD (Augustinack et al., 2002; Andrade-Guerrero et al., 2023). The mechanism for the initiation of hyperphosphorylation of these tau proteins is not known; however, the deposition of Aβ fragments has been suggested to act as an upstream pathophysiological event triggering other AD-associated pathogenic events especially the NFTs.

In the CNS of humans, apolipoprotein E (ApoE), existing in three isoforms ApoE2, ApoE3, and ApoE4, is involved in the synthesis and transport of cholesterol (Bu, 2009). A single amino acid difference in the 299-amino acid-long ApoE (all isoforms) can alter its structure and influence functional abilities (Frieden and Garai, 2012). These isoforms have varying pathological properties in AD in the order ApoE4 > ApoE2 > ApoE3 (Serrano-Pozo et al., 2015; Serrano-Pozo et al., 2016; Mahoney-Sanchez et al., 2016). *In vitro* studies on human cell lines and induced pluripotent stem cells (iPSCs) have indicated that APOE4 may stimulate the AD pathology including tau phosphorylation (Wang C. et al., 2018). Although several studies have been conducted to understand the functional ability of ApoE variants, more research in this field is required for a better comprehension of how distinct ApoE isoforms affect Aβ aggregation and clearance in AD pathogenesis (Kim et al., 2009; Husain et al., 2021; Serrano-Pozo et al., 2021).

Besides the abovementioned proteins, enzymes, and genes, a special type of glial cells, known as microglia, plays an important role in AD pathogenesis. These cells are immune components of the CNS, which are activated and concentrated around the Aβ plaques or NFTs during AD progression and help in their clearance. However the hyperactivation of microglial cells leads to neurotoxicity and promotes Aβ production (Wang et al., 2015; Hansen et al., 2018). Molecular changes in AD are not only restricted to Aβ and NFTs, but various other and associated changes occur during AD progression; these include damages to synapses, neurotransmitters, neuronal organelles, such as mitochondrial and endoplasmic reticulum, and ubiquitin proteasome system, histone modification, altered protein synthesis, altered cell cycle, and cell death (Arendt, 2005; Ferrer, 2012; Ferrer, 2022). Furthermore, various genes, including *ABCA7*, *BIN1*, *CASS4*, *CELF1*, *CD33*, *CD2AP*, *CELF1*, *BIN1*, *PICALM*, *EPHA1*, *SORL1*, *CR1*, *EPHA1*, *HLA*, *IL1RAP*, *INPP5D*, *MS4A*, *TREM2*, and *TREM2L*,are directly or indirectly involved in the pathogenesis of AD (Carrasquillo et al., 2009; Seshadri et al., 2010; Karch and Goate, 2015; Ramanan et al., 2015; Dorszewska et al., 2016).

There are usually two common types of Alzheimer’s disease including familial Alzheimer’s disease (FAD) and sporadic Alzheimer’s disease (SAD) (Jojo et al., 2025). FAD is a rare (less the 1% of total cases) genetically inherited (autosomal dominant) form that typically presents at an earlier age, often before 65, and is linked to mutations in specific genes such as *APP*, *PSEN1*, and *PSEN2*. These genetic mutations lead to abnormal processing of amyloid precursor protein and the accumulation of amyloid plaques, which are key features of Alzheimer’s pathology (Dorszewska et al., 2016; De Deyn and Sleegers, 2025; Ehn et al., 2025). On the other hand, SAD is far more prevalent and usually occurs after the age of 65 and hence also known as late onset Alzheimer’s disease (LOAD). The onset of SAD is influenced by a combination of genetic, environmental, and lifestyle factors. The *APOE* ε4 allele is the most notable genetic risk factor for SAD (Akhtar et al., 2024; Jojo et al., 2025).

### 4.2 Sex differences in the prevalence of AD

Sex may have a significant impact on the onset and course of AD. However, a clear report or explanations on sex-based differences in the AD prevalence are not available. Studies have found contradictory results on sex differences, either showing no sex differences or faster annual rates of normal cognitive loss (age induced) in men and women (Wiederholt et al., 1993; Barnes et al., 2003; Proust-Lima et al., 2008; Mielke et al., 2014). Similar results have been found regarding mild cognitive impairment (MCI), with some studies showing a higher prevalence in men (Koivisto et al., 1995; Ganguli et al., 2004; Petersen et al., 2010), whereas others showing prevalence in women (Larrieu et al., 2002; Di Carlo et al., 2007) or no sex-based differences (Kivipelto et al., 2001; Solfrizzi et al., 2004). Similar to cases of cognitive decline and MCI, the prevalence studies related to sex differences in actual AD cases are ambiguous. Reports suggest the incidence of actual AD cases is much greater in women than in men (Hebert et al., 2013). According to some reports, women make up nearly two-thirds of those with an AD diagnosis. However, contradictory reports suggest higher incidence of AD cases in men until age 78 and after which females had higher incidences (Miech et al., 2002). Consistent with studies by Miech and his group, the rate at which MCI progresses to AD has been shown to be comparable for men and women between the ages of 70 and 79, but it is higher for women than men after the age of 80 (Roberts et al., 2014). The majority of research on Asian and European populations has found that women aged more than 80–85 years have a higher incidence of AD. Hence, although numerous studies have been done on the sex differences in AD prevalence, it remains unclear whether there are actually sex differences or not. However, majority of studies suggest that women are more likely to develop AD than men. Although the exact cause is not known and the pathways and risk factors can differ, a possible explanation could be based on the fact that women live longer than men, leading to increased incidence among men (Seshadri et al., 1997; Plassman et al., 2007); women being at higher risk of anxiety and depression compared to men (Kessler et al., 1993; Mielke, 2018); women having fewer opportunities for higher education (Karp et al., 2004; Langa et al., 2017; Russ et al., 2013) and being subject to pregnancy, menopause, and hormonal changes (Mielke et al., 2016). These observations may account for some of the variations in the risk, despite the fact that they might be stereotyped and not always accurate.

## 5 Challenges in studying and treating CNS-related disorders

CNS related disorders are life threatening and difficult to treat because of the complexity of the brain structure, which contains various cell types. The primary obstacle that remains to be addressed in managing CNS disorders is the absence of global gene expression data for the brain (Do Thi et al., 2004). Another challenge in treating neurological disorders is the limited access to brain structures because of the presence of a physical barrier, the BBB (Hocquemiller et al., 2016; Scarpa et al., 2017; Ganipineni et al., 2018). Furthermore, the physiological and pharmacological challenges in gene therapy associated with various delivery systems encountered in therapeutic targeting of the brain because of low BBB permeability and brain structure complexity have been reviewed earlier (Joshi et al., 2017). Among the various routes of administration, such as intranasal, intracarotid, intravitreal, intrathecal and intramuscular, the intravenous and intracerebral administration methods are considered the most effective (Cwetsch et al., 2018). Even though the intracerebral route of administration provides a focused/targeted method, diffusion of drugs into unwanted areas makes it unfavorable at times (Mali, 2013). Another challenge is to extend the *in vitro* findings to in vivo research (for example, from mice to primates) in gene therapy and the design of the delivery vehicle varies among models (Gray et al., 2011; Joshi et al., 2017).

## 6 Role of CRISPR/Cas9 in AD

CRISPR/Cas9 has been used to create cellular and animal models of a number of neurological conditions, which enables researchers to better understand disease mechanisms and seeking answers to the underlying causes of serous neurological conditions, such as AD (Schrauben et al., 2020), PD (Yang et al., 2019), and autism (Elamin et al., 2023). Recent studies have suggested CRISPR/Cas9 as a promising genome editing approach for modeling AD and therapeutic treatment of AD (Rohn et al., 2018; Barman et al., 2020; Lu et al., 2021; Bhardwaj et al., 2022; De Plano et al., 2022; Chacko et al., 2023). This technique has been used for therapeutic purposes in both early-onset and sporadic AD models (Barman et al., 2020).

The CRISPR/Cas9 technology has been employed extensively in the field of AD in recent years for target therapy, pathogenic gene screening, and creation of AD models because of its short experimental duration (Chacko et al., 2023). Numerous *in vitro* cell models of AD have been created in the last few decades. Human neuroblastoma cells, SHSY5Y and SK-N-SH, mouse hippocampus neuronal cell lines, HT22 and glial cells, BV2, mouse glioblastoma cells, N2a, and other cells are often used in AD research. Both early-onset and sporadic AD models can benefit from this therapy approach. CRISPR may efficiently target any specific gene sequence to fix mutations and to add genetic elements to the targeted DNA regions in cells or tissues (Swiech et al., 2015). Better cellular and molecular replicas, functional knockout, investigations of lethal neuronal damage, disease modeling, and genome insertion of the guide gene sequence have been effectively achieved using this tool. Additionally, this technology has the potential to treat genetic disorders related to CNS, and researchers have been investigating its potential to correct mutations linked to neurodegenerative diseases, such as Huntington’s disease (Morelli et al., 2023), spinal muscular atrophy (Zhou et al., 2018), and some forms of epilepsy (Colasante et al., 2020).

### 6.1 Models of AD created using CRISPR/Cas9

The majority of AD models were created by overexpressing human mutant genes that produce tau and Aβ. Although these models have shown great promise in comprehending certain facets of AD pathogenesis, their applicability is limited because the majority of AD animal models do not exhibit overt neurodegeneration (LaFerla and Green, 2012). Various models have been generated using the CRISPR/Cas9 technology, which displaying a more accurate disease phenotype in various cell lines, such as stem cells, iPSCs, and transgenic animals (including rat, mice, swine), as summarized in Table 2.

**TABLE 2:** Applications of CRISPR/Cas9 technology in AD in generation various *in vitro* and *in vivo* AD models.

Gene/protein target	Mutations	Cell line/animal	Results	References
APP	APPSw (knock-in)	Human iPSCs	Altered Aβ metabolism	Paquet et al., 2016; György et al., 2018
	C-terminus	iPSCs, cultured neurons, mouse brain	Attenuated β-cleavage and Aβ production	Sun et al., 2018
	G676R, F681Y, and R684H	(App*^hu/hu^*)rat and mouse	Decreased BACE1-dependent processing of APP	Serneels et al., 2020
‘PSEN1	M146V (knock-in)	Human iPSCs	Altered Aβ metabolism	Paquet et al., 2016
	138 different mutations (knock-out)	N2a	Affects γ-secretase Aβ42/40 ratio	Sun et al., 2017
	S1R (knock-out) S1R (knock-in)	Transgenic mouse	Fewer mushroom-shaped dendritic spines Higher number of mushroom-shaped dendritic spines	Maurice et al., 2019; Ryskamp et al., 2019
PSEN2	N141	iPCScs	Increased Aβ42/40 ratio	Ortiz-Virumbrales et al., 2017
BACE1	5XFAD (along with APP knock-in)	Transgenic mice	Altered Aβ metabolism	Park et al., 2019
Txnip	Downregulated	HT22	Attenuated Aβ-induced cysteine oxidative modification	Wong et al., 2019; Lu et al., 2021
KIBRA	Downregulated	HT22	Apoptosis, while treating with Aβ(1–42)	Song et al., 2019
PSEN1	Knockdown	N2a	Decreased production of Aβ	Sun et al., 2017
Rag1 and Ppar-γ	Gene modifications	Monkeys	–	Niu et al., 2014; Chen et al., 2015

APP, amyloid precursor protein; PSEN1, Presenilin 1; PSEN2, Presenilin 2; Txnip, Thioredoxin-interacting protein; KIBRA, Kidney and BRAin expressed protein; BACE, beta-site amyloid precursor protein cleaving enzyme; Plcγ2, phospholipase C gamma 2.

The CRISPR/Cas9 technology has also been widely used to create disease models for neurodegenerative diseases, such as PD, Huntington’s, and ALS, which mimic human conditions by editing genes known to be implicated in these disorders. In PD, CRISPR/Cas9 is used to generate models with mutations in the *LRRK2* and *SNCA* genes, providing insights into the development of alpha-synuclein aggregates and dopaminergic neuron loss. Similarly, CRISPR/Cas9 has been applied to introduce expanded CAG repeats in the *HTT* gene, enabling the creation of cellular and animal models that recapitulate the key features of Huntington’s disease, offering insights into potential therapeutic approaches aimed at reducing toxic protein aggregates. CRISPR/Cas9 has also been used to study psychiatric disorders, such as schizophrenia, depression, and autism, where genetic and environmental factors interact in complex ways. For example, CRISPR/Cas9 has facilitated the creation of Schizophrenia models with mutations in *DISC1*, *NRXN1*, and *CACNA1C*. Similarly, genetic mutations in the *SHANK3* gene and genes encoding other synaptic proteins have been studied using CRISPR/Cas9 to model ASD (Nojadeh et al., 2023).

## 7 Role of CRISPR/Cas9 in targeting important AD biomarkers

This section mainly discusses the gene-based therapeutic strategies for AD, which focus on the regulation of Aβ and Tau expression using the CRISPR/Cas9 technology alongside targeting various other biomarkers of AD. Various genes, proteins and enzymes are targeted using CRISPR/Cas9 technology in numerous preclinical studies as shown in Table 3 are discussed as under.

**TABLE 3:** Various approaches of using of CRISPR/Cas9 technology to study various AD models and its possible therapeutic role.

Gene/protein	Mutations	Cell line or animal	Result	References
APP	APP^Swe^ (Knock out or deletion)	Fibroblasts	Abrogates Aβ formation; reduction in secreted Aβ	Mullan et al., 1992; György et al., 2018
		Tg2576 mice	Remains unstudied for Aβ pathology	György et al., 2018
	deletions within 700 bp in 3’-UTR of APP	Mice (APP-KI)	Reduction in Aβ pathology	Nagata et al., 2018
	C-terminus	HEK293, ATCC	reduced Aβ pathology	Sun et al., 2018
	C terminus of APP	iPSCs and Mouse	Downregulating β-cleavage and up-regulating α-cleavage leading to reduced Aβ	Sun et al., 2019
	C99 fragment (T48P, L52P, K53N)	HTL cells	Reduced Aβ40 cleavage by γ-secretase	Xu et al., 2016
APOE4	Conversion of APOE E4 to E3/E2	iPSCs	Prevents the pathology related to APOE4	Komor et al., 2016; Rohn et al., 2018; Wang C. et al., 2018
PSEN2	PSEN2^N141I^	iPSCs	Reduction in Aβ levels; Normalization of the Aβ 42/40 ratio.	Pires et al., 2016; Poon et al., 2016; Ortiz-Virumbrales et al., 2017; Moreno et al., 2018
*BACE1*	5XFAD	Mice	Significant decline in BACE expression and beta cleavage products of APP	Park et al., 2019
MAPT	Deletion in codon 1	Mice (tauΔex1)	No excitotoxicity No memory loss	Tan et al., 2018
Plcγ2	Plcγ2-P522R	Mice (knock-in)	Increases microglial function and hence reduces the risk of AD	György et al., 2018

APP, amyloid precursor protein; APOE4, apolipoprotein E4; PSEN2, presenilin 2; BACE, beta-site amyloid precursor protein cleaving enzyme 1; MAPT, microtubule associated protein tau); Plcγ2, phospholipase c gamma 2; iPSCs, induced pluripotent stem cells; Aβ, amyloid beta; AD, Alzheimer’s disease.

### 7.1 APP

CRISPR/Cas9 has been employed to introduce mutations in APP that can lead to the production of toxic Aβ peptides. These models help in understanding the molecular cascades that contribute to neurodegeneration and in testing potential therapeutic interventions. APP is a key target protein in for AD because it can be cleaved and processed by a variety of enzymes to produce either soluble or pathogenic Aβ peptides. For example, the KM670/671NL APP mutation causes β-secretase to cleave the molecule more frequently, which increases Aβ levels (György et al., 2018). Studies have also shown that knocking out the *APP* gene using CRISPR/Cas9 can decrease the expression of Aβ, suggesting its role in gene therapy. Moreover, deletion mutations in the 3′-untranslated region (UTR) of mice *App* gene could cause reduction in Aβ accumulation (Nagata et al., 2018). Another example highlighting the role of APP and CRISPR is from an Icelandic population that did not exhibit AD symptoms at advanced ages due to A673T mutation. This mutation results in a 40% decrease in β-secretase cleavage in such population. In the light of this finding, scientists postulated that introducing this mutation into patients’ neurons would be a viable and efficient strategy to impede or even slow the progression of AD. Accordingly when this mutation was successfully introduced in 53% of HEK293T cells together with a new mutation (E674K), the accumulation of Aβ peptide was significantly reduced (Guyon et al., 2021). Studies have also shown that selectively altering the endogenous APP at its C-terminus in both animal and cell models using the CRISPR technology reciprocally altered the amyloid pathway leading to a decline in APP-β-cleavage and Aβ synthesis (Sun et al., 2019).

### 7.2 BACE1

The sequential processing or modification of APP is carried out by γ-secretase along with BACE1 protein to produce Aβ protein (Park et al., 2019). Therefore, targeting BACE1 is a potential therapeutic approach for AD (Lu et al., 2021). The rates of enzyme activity and BACE1 concentration can be potential biomarkers in therapeutic studies evaluating the role of BACE1 inhibitors in regulating APP processing. The targeting of the *Bace1* gene was reported to suppress Aβ-associated pathologies and cognitive deficits in mouse models of AD (Park et al., 2019). Hence, the increased expression of BACE1 can serve as an early biomarker for AD detection (Blennow et al., 2010; Evin et al., 2010).

### 7.3 Gamma (γ)-secretase

γ-Secretase, a large intermembrane protein complex regulated by γ-secretase activating protein (GSAP), is another target for gene therapy of AD. The reduction in GSAP expression can dramatically lower the Aβ levels (He et al., 2010; Ghosh and Surolia, 2011). Furthermore, knocking out GSP in HEK293 cells stably expressing APP (HEK-APP) using the CRISPR/Cas9 technology caused a significant decline in Aβ secretion and γ-secretase activity (Wong et al., 2019).

### 7.4 PSEN1/2

Both the PSEN1 and PSEN2 are essential components of the γ-secretase complex, the activity of which is controlled by GSAP expression. Hence, PSEN1/2 mutations are associated with AD, especially to the majority of familial AD cases (Raux et al., 2005; Wu et al., 2011; Chávez-Gutiérrez et al., 2012; Wang P. et al., 2018). The majority of PSEN1 mutations affect amyloid metabolism, which raises the Aβ42/40 ratio as well as Aβ42 levels and lowers the generation of Aβ1–40 (Hung and Livesey, 2018; Sasaguri et al., 2018). Additionally, the majority of early-onset familial AD have been confirmed to be caused by mutations in the *PSEN1* gene. In one such study, a Chinese familial AD pedigree revealed a unique V97L missense mutation at codon 97 (Val97Leu) of the *PSEN1* gene. The CRISPR/Cas9 technology was used to create a mutant SH-SY5Y cell line in which Aβ production was significantly increased (Fang et al., 2006), suggesting that PSEN1 mutations have a role in AD pathophysiology.

Similarly PSEN2 mutations can lead to AD pathology. Cells harboring an N141I mutation in *PSEN2* exhibited significantly higher Aβ42/40 concentrations. Rectify of this mutation in iPSCs derived from neurons using CRISPR/Cas9 resulted in normalized Aβ42/40 ratio (Ortiz-Virumbrales et al., 2017).

### 7.5 APOE

The astrocytes in the CNS are the primary source of APOE. Neuronal APOE expression has been implicated in age-related cognitive decline, neurological damage, and neurodegeneration. A change of the E4 allele of APOE to E3/E2 using the CRISPR/Cas9 technology resulted in a significant decline in tau phosphorylation in neurons and made them less vulnerable to cytotoxicity (Wadhwani et al., 2019). Furthermore, the role of APOE4 in Aβ metabolism has been highlighted in studies using human IPSCs (hiPSCs) and CRISPR/Cas9 technology (Lin et al., 2018). Moreover, some AD-related disorders could be attenuated by isogenic conversion of APOE4 to APOE3 (Huang et al., 2017). These results suggest APOE4 as one of the most potent risk factor and good target in AD therapy.

### 7.6 CD33

Human genetic association studies suggest the involvement of immune response in AD etiology. Neutrophils and microglia express CD33, an immunomodulatory receptor, at different levels. It plays different roles in controlling phagocytosis, which it’s causatively related to AD pathology (Griciuc et al., 2013; Estus et al., 2019). mCD33 genetic ablation in U937 (with high hCD33Mexpressing) using the CRISPR/Cas9 technology was reported to reduce the clinical phenotype of AD and improved microglia phagocytosis to promote Aβ clearance (Bhattacherjee et al., 2019). Additionally, these researchers used CRISPR/Cas9 to damage the *CD33* gene and complemented it with several hCD33 variations, which reduced Aβ pathology and neurodegeneration. Another variant of hCD33, hCD33m, was reported to enhance Aβ phagocytosis and suppresses phagocytosis (Bhattacherjee et al., 2021). These findings strongly suggest the possibility of using CD33 receptors in the treatment of AD.

### 7.7 GMF

A recently identified proinflammatory protein called glial maturation factor (GMF) is strongly expressed in different areas of the AD brain and is mostly expressed in the reactive glial cells that surround the amyloid plaques (Ahmed et al., 2017). Overexpression of GMF activates the p38 MAPK signaling pathway and causes oxidative damage resulting in the death of neuronal cells. The CRISPR/Cas9 technique was used to decrease GMF expression in BV2 cells, which inhibits pp38 MAPK to control GMF-induced proinflammation in microglia (Raikwar et al., 2019).

### 7.8 Cys-LT1R

Two important G-protein coupled receptors (CysLT1R and CysLT2R) trigger inflammatory signaling cascades in response to a class of inflammatory lipid molecules known as cysteinyl leukotrienes (Cys-LTs). Accumulating evidence indicates that CysLT1R is closely linked to the onset and progression of AD and can mediate the inflammatory response via the NF-kB pathway (Yu et al., 2005; Wang et al., 2011). An increase in CysLT1R expression was reported to be associated with high levels of Aβ1-42 and an antagonist of this receptor could significantly inhibit the overexpression of CysLT1R-mediated inflammatory response via the NF-kB pathway. Hence, the deletion of CysLT1R using the CEISPER/Cas9 technology can reduce Aβ pathology (Chen et al., 2021).

## 8 Important parameters related to CRISPR/Cas9 and its role in CNS-related disorders

While several functional capabilities of CRISPR/Cas9 point to its role and significance in a range of CNS-related therapeutic approaches, the three most crucial ones are described below.

### 8.1 Target discovery and validation

CRISPR/Cas9 can be used for high-throughput genetic screens within the CNS. By knocking out or modulating the expression of thousands of genes in parallel, CRISPR screens can identify key regulators of CNS disease pathways. Several studies have used such screens to uncover novel targets that drive neurodegeneration or psychiatric symptoms (Shalem et al., 2015; Kampmann, 2020; Jiang et al., 2024).

### 8.2 High-throughput CRISPR screens

Using CRISPR libraries, genes in neuronal or glial cells can be systematically knocked out to assess their role in disease. For example, CRISPR screens have been applied to identify novel modulators of neuroinflammation, which play crucial roles in AD and other neurodegenerative diseases. These screens help in identifying potential drug targets that could ameliorate disease progression (Duan et al., 2021a; Sanchez et al., 2021; Saurat et al., 2024).

### 8.3 Functional validation of CNS disease targets

After potential targets are identified, CRISPR/Cas9 can be used to validate their role by editing these genes *in vivo*. In CNS disease models, the functional consequences of gene knockout or mutation can be studied in terms of neuronal viability, synaptic function, and behavioral outcomes, providing crucial data for developing therapeutic strategies (Gotz et al., 2018; Mani et al., 2023; Wang et al., 2024; Akyuz et al., 2024).

## 9 Clinical trials for Alzheimer’s disease

Although there are currently no clinical trials using CRISPR/Cas9 for Alzheimer’s disease (AD), this gene-editing technology holds significant potential for targeting genes that regulate amyloid beta (Aβ) and tau proteins, two key pathological features of AD. Currently, these gene-editing approaches are in the preclinical stage, with research primarily focused on animal models to evaluate their safety and effectiveness before advancing to human trials. CRISPR could be used to directly edit the genetic mutations that lead to the production of toxic Aβ plaques or abnormal tau tangles, potentially preventing or slowing the progression of the disease. By precisely modifying the genes responsible for these protein aggregations, CRISPR has the promise of offering a more targeted and long-term therapeutic approach, moving beyond symptom management to addressing the root causes of Alzheimer’s. However, translating these findings into clinical applications will require overcoming significant technical and safety challenges. Although we did not find any information about the clinical trials using CRISPR/Cas9 in AD but a number of drugs are currently being used in various stages of clinical trials.

### 9.1 Aducanumab

It is a monoclonal antibody sponsored by Biogen and designed to target and clear amyloid plaques. It was approved by the FDA in 2021 but its effectiveness remains debated. Clinical trials suggest that it can reduce amyloid plaques but only modestly impacts cognitive decline. Using this drug multiple phase-3 trials have been conducted, including the *EMBARK* and *PRIME* studies (Vaz et al., 2022; Heidebrink and Paulson, 2024; Kim et al., 2025).

### 9.2 Lecanemab

Approved in 2023 and sponsored by Eisai and Biogen, these are monoclonal antibodies sponsored by Eisai and Biogen that target amyloid-beta plaques. It is an ongoing clinical trial currently in phase-3 stage. It has shown promises in slowing the progression of AD during early stages. *Clarity AD* phase-3 trial demonstrated its ability to reduce amyloid plaques and slow cognitive decline (Park et al., 2024; Schiller et al., 2024; Arroyo-Pacheco et al., 2025).

### 9.3 Donanemab

This is an antibody sponsored by Eli Lilly that specifically targets a modified form of amyloid known as *N3pG*. Early trials have shown some promising results, especially in patients with early-stage Alzheimer’s. It is in ongoing phase-3 clinical trial stage (Mintun et al., 2021; Kim et al., 2025).

### 9.4 Tilavonemab

It is an antibody designed to target tau tangles in the brain. The trial is evaluating its effectiveness in slowing disease progression in Alzheimer’s patients. This drug is in ongoing phase-2 trial stage (Florian et al., 2023; Cai et al., 2025).

### 9.5 LMTX

It’s a novel formulation of Methylene blue, a compound with antioxidant and neuroprotective properties and can target Tau aggregation. This drug was sponsored by TauRx Therapeutics and previous trials have shown mixed results, but further research is being conducted to explore its potential benefits. Currently, it is in phase-3 trial stage (Baddeley et al., 2015; Congdon et al., 2023).

### 9.6 Simufilam

It is a drug sponsored by Cassava Science that works by modulating the abnormal tau protein and reducing neuroinflammation. It has shown early positive results in slowing cognitive decline and is currently undergoing larger trials under phase-3 (Burns et al., 2023; Tartaglia and Ingelsson, 2025).

### 9.7 Tanezumab

It is a monoclonal antibody sponsored by Pfizer targeting nerve growth factor (NGF), a protein that plays a role in neurodegeneration. It has been tested for its potential to treat pain in other conditions and is now being explored in Alzheimer’s.

A combination of *Memantine* (a glutamate regulator) and *donepezil* (a cholinesterase inhibitor) is used to treat moderate-to-severe Alzheimer’s (Zeng et al., 2025). Other than this, some trials are investigating the combination of amyloid-targeting agents like *Aducanumab* and *Lecanemab* with other treatments like *tau modulators* or *anti-inflammatory drugs* to see if a multi-pronged approach is more effective (Almutairi, 2025).

## 10 Challenges in CRISPR/Cas9-based strategies against AD

Despite gene therapy-based techniques with potential to reverse neurodegenerative disorders including AD, several challenges, including efficient delivery across the BBB, ensuring specificity, and avoiding off-target effects, remain a hurdle in practical implementation. The application of CRISPR/Cas9 technology in AD is challenging because most of the AD cases are sporadic and have various unidentified causes (Bettens et al., 2013). While CRISPR/Cas9 presents a promising avenue for correcting genetic mutations associated with early-onset familial AD, it is important to recognize that these cases account for less than 1% of all AD cases (Jojo et al., 2025). The vast majority of AD cases are sporadic and arise from complex interactions between genetic risk factors (such as APOE ε4), environmental influences, and aging-related processes (Akhtar et al., 2024; Jojo et al., 2025). Since CRISPR-based interventions target specific gene mutations, they do not currently address the multifactorial and largely unknown etiology of sporadic AD. As a result, the broad application of CRISPR therapeutics for AD remains challenging, and further research is needed to explore its potential role beyond monogenic forms of the disease.

Another challenge is the uncertainty in identifying, diagnosing, and treating the patients during early stages of disease progression (Isik, 2010). Furthermore, gene transport to the target areas of cells may be ineffective because it can cause mutation in off-target regions, which can affect the functionality of altered cells (O’Geen et al., 2015). Moreover, the treatment for AD involves a variety of efforts to reach the impacted cells in the CNS, which adds extra challenges because of the structural complexity, biological diversity, and inability to cross the BBB (Mikkelsen et al., 2019).

Despite the development of numerous viral and nonviral vectors, effective and targeted gene substitution is difficult to achieve, and the best delivery methods still need to be devised. The practice of altering genes in post-mitotic cells under *in vivo* conditions is in experimental stages. Virus-mediated CRISPR/Cas9 editing is also limited by the integration of the carrier genome (virus vector) into the host genome, inducing immunogenic responses (Crudele and Chamberlain, 2018). Advances in delivery systems, such as nanoparticles-based carriers and improved viral vectors, are critical to translating CRISPR into clinical therapies for CNS diseases (Zhou Y. et al., 2020).

### 10.1 Bioethical concerns, regulatory approvals, and safety considerations

While CRISPER/Cas9 holds significant potential for targeting genes linked to Alzheimer’s disease (AD), its clinical application raises a number of bioethical, regulatory, and safety concerns (Rafii et al., 2022; Rahimi et al., 2024; Zhai, 2024).

### 10.1.1 Germline editing

One of the biggest ethical concerns is the potential for germline editing, where changes are made to the DNA of embryos or germline cells. This could lead to unintended and permanent genetic consequences that are passed down through generations with potential unknown long-term consequences (Lanphier et al., 2015; Barman et al., 2020).

### 10.1.2 Social implications

The potential to “edit” genetic material may raise concerns about the possibility of genetic discrimination with implications for insurance issues, or even the creation of genetic “haves” and “have-nots,” especially in terms of access to cutting-edge therapies. Hence, these concerns remain particularly relevant in the clinical use of gene editing for AD, where access to advanced therapies could become a societal issue (Rafii et al., 2022).

### 10.1.3 Consent

With therapies like CRISPR, the issue of informed consent becomes complex, especially when considering vulnerable populations like elderly or those affected by AD. It is crucial that AD patients and their families fully understand the potential risks of CRISPR/Cas9 based therapies (Doudna and Charpentier, 2014; Rafii et al., 2022).

Other than the above-mentioned issues, the introduction of the CRISPR/Cas9 system into the body could trigger an immune response, as the Cas9 protein may be recognized as foreign by the immune system (Crudele and Chamberlain, 2018; Charlesworth et al., 2019). Furthermore, Off-target mutations remain a significant risk. Even small errors in gene editing could lead to unintended genetic changes, potentially causing harmful effects. In addition, editing embryos raises moral questions about whether human embryos should be subject to experimental genetic modification (Lanphier et al., 2015). CRISPR-based therapies being expensive can cause ethical concerns related to healthcare disparities where only wealthy individuals can afford the treatment (Tjandrawinata et al., 2025). While CRISPR/Cas9 presents a revolutionary approach to genetic interventions for Alzheimer’s disease, ensuring it’s ethical, regulatory, and safety compliance remains paramount before widespread clinical application.

### 11 Alternative gene-editing tools other than CRISPR

While CRISPR/Cas9 has been a transformative tool in gene editing, alternative technologies such as base editing, prime editing, and zinc-finger nucleases (ZFNs) have also emerged, offering distinct advantages in terms of precision and targeting specific mutations, particularly in the context of Alzheimer’s disease (AD) (Naeem et al., 2020). Some of the important alternative gene-editing techniques are;

### 11.1 Base editing

It allows the direct conversion of one DNA base pair to another without inducing double-strand breaks, thus minimizing the risk of unwanted mutations and off-target effects, making it particularly useful for correcting point mutations associated with AD (Naeem et al., 2020; Guyon et al., 2021). As many of the mutations linked to AD, such as those in the APP, PSEN1, and PSEN2 genes, often involve specific point mutations that could potentially be corrected at the DNA level using base editing (Sasaguri et al., 2018; Naeem et al., 2020). For example, base editing could be employed to precisely convert a mutated base in the APP gene responsible for amyloid-beta production, thereby halting the formation of toxic amyloid plaques (Guyon et al., 2021).

### 11.2 Prime editing

Often regarded as more accurate than CRISPR/Cas9, enables precise and versatile editing of genetic sequences, including the potential for complex edits, without relying on double-strand breaks (Ochoa- Sanchez et al., 2021). However, its application is still in early stages and is less widespread (Rottner et al., 2025). While CRISPR/Cas9 relies on introducing double-strand breaks and the cell’s repair mechanisms to make edits, prime editing utilizes a modified Cas9 protein that introduces a single-strand break and a reverse transcriptase enzyme to directly insert the desired genetic changes without requiring double-strand breaks. This method can thus allow for more accurate and efficient editing, with fewer off-target effects, making it especially valuable for correcting the subtle genetic mutations associated with AD, such as those in the APP or PSEN1 genes (Chen and Liu, 2023; Rottner et al., 2025).

### 11.3 Zinc-finger nucleases

Zinc-finger nucleases (ZFNs) are one of the earliest forms of gene-editing tools and have been explored in AD research for their ability to target and modify specific genes (Shippy and Ulland, 2022). ZFNs function by fusing a DNA-binding zinc-finger protein domain with a nuclease domain that induces double-strand breaks at targeted locations, enabling the correction of genetic mutations. However, ZFNs have some limitations, such as off-target effects and challenges in designing specific zinc-finger domains for new target sequences (Zhang, 2024). While CRISPR/Cas9 remains the most widely used tool due to its efficiency and broad application, the above-mentioned gene-editing technologies provide complementary options that may offer higher precision for correcting mutations linked to AD, particularly in situations where fine-tuned edits are required (Chehelgerdi et al., 2024; Li et al., 2024; Bolideei et al., 2025).

## 12 Role of nanotechnology in CRISPR/Cas9-based treatment of AD

The abovementioned obstacles pose significant difficulties in the therapeutic application of CRISPR/Cas9. Numerous viral and nonviral nanovectors have been used to effectively overcome the obstacles. Different strategies to combat the shortcomings of CRISPR/Cas9 delivery systems include development of various nanoparticles-based vectors called as nanovectors, including nanocomplexes, nanoclews, nanoassemblies, and gescicles. Although viral nanovectors effectively solve the delivery related problems, other challenges including low cargo-loading capacity, long production and purification times, poor immuno- and bio-compatibility, and poor biocompatibility frequently restrict their use as delivery vectors (Xu et al., 2019; Wei et al., 2020). Carefully designed nonviral nanovectors are not only safer, but biocompatible and immunocompatible and they can effectively traverse the BBB to transport the cargo via extracellular barriers to reach therapeutic targets both at cellular and nuclear levels.

Typical nanocomplexes are created by electrostatically attaching cationic polymers, peptides, or lipids to the negatively charged CRISPR/Cas 9 system. The Cas9 enzyme and gRNA are encapsulated within the nanocomplex with improved cellular absorption, and protection from cellular degradation (Park et al., 2019). Nanoclews are complexes made of DNA and polyethylenimine. CRISPR/Cas 9 is delivered to the nanoclew by chemical conjugation or noncovalent interactions between the Cas9 enzyme and gRNA. Compared to traditional nanostructures, which are lengthy and extremely complex, nanoclews offer a number of advantages. Given the biocompatibility and biodegradability of the DNA scaffold used in nanoclews, they can be readily altered to target particular cells or tissues, reducing toxicity and immunological reaction (Sun et al., 2015; Hanafy et al., 2020). For targeting certain cells or tissues, nanoclews can be embellished with targeting molecules, such as peptides and antibodies. Gescicles are nanovesicles for CRISPR/Cas9, which are composed of lipids and proteins derived from cell membranes of RBCs and glycoprotein of vesicular stomatitis virus G. These nanovesicles are injected into the bloodstream with loaded CRISPR/Cas9 to be delivered at target cells/tissues. Gescicles have several advantages over other nanocarriers. They are biocompatible and biodegradable. They may target particular cells or tissues upon alteration of their surface proteins, have a high payload capacity (can carry a significant amount of CRISPR/Cas 9), and do not cause any severe immune response (Scopa et al., 2020).

Magnetic nanoparticles can be used for delivering CRISPR/Cas 9 to target cells. In order to precisely target cells of interest, magnetic nanoparticles can be functionalized with targeting moieties, such as peptides and antibodies. They can also be magnetically steered to their target site for more precise drug administration. The delivery of CRISPR/Cas 9 via magnetic nanoparticles is possible by the formation of a magnetofection complex. The CRISPR/Cas 9 components are attached to the surface of magnetic nanoparticles to generate these complexes. Magnetisable particles coated with CRISPR/Cas9 components and an external magnetic field can then be used to drive the complexes to the target cells. The cells absorb these particles, and an external magnetic field directs them to the nucleus (Mout and Rotello, 2017). One benefit of delivering CRISPR/Cas 9 via magnetic nanoparticles is that they offer a non-invasive approach for targeting cells *in vivo*, potentially lowering the possibility of adverse consequences from off-target delivery. Furthermore, imaging methods like magnetic resonance imaging can be used to track magnetic nanoparticles, enabling real-time therapy and monitoring of the administered medication (Ribarič, 2021).

Another type of nanoparticle-based carriers comprises positively charged lipids that can easily combine with the CRISPR/Cas9 system owing to its anionic nature to form complexes known as lipoplexes. In this system, the enzyme (Cas9) and the RNA segment (gRNA) are contained within a lipoplex to administer CRISPR/Cas9. Furthermore, by altering its surface with different particles (targeting moieties), such as peptides and antibodies, lipoplex can be directed to particular cells or organs. A benefit in employing lipoplexes for CRISPR/Cas9 delivery is their ease of preparation and scalability for large-scale manufacturing. Furthermore, lipoplexes prevent the CRISPRCas9 system from degrading and promote its cellular absorption, which can increase the effectiveness of delivery and therapeutic efficacy (Campbell et al., 2019).

Although the delivery techniques based on nanoparticles mentioned above offer a better chance of delivering CRISPR/Cas9, further studies are required to determine the safety and efficacy of these nanoparticles as well as to maximize their therapeutic effect and efficacy. Despite the several advantages, each of these methods has certain drawbacks. For instance, the cationic polymers (lipids or proteins) utilized in nanocomplexes and lipoplexes may cause an immunological reaction, whereas the DNA scaffold employed in nanoclews may also elicit the same and can induce cytotoxicity. Furthermore, several intrathecal and intracerebroventricular injections are required to achieve adequate therapeutic distribution in the brain as systemic administration of nanocomplexes is ineffective at delivering cargo across the BBB. Gescicle research is still in its infancy, and further studies are required to determine their effectiveness and safety. Similarly, the heat produced by the strong magnetic field gradients needed for magnetic nanoparticle targeting may damage cells. Furthermore, issues with magnetic nanoparticles, such as clearance, biocompatibility, and immunological reactions, may arise under *in vivo* conditions (Ribarič, 2021). Currently researchers in this frontier area are working to address current shortcoming to offer efficient and safer delivery systems for therapeutic intervention.

## 13 Future prospects for CRISPR/Cas9 in AD

Recent use of artificial intelligence (AI) and deep learning computational algorithms has made considerable progress in gene editing, improving its precision and efficiency by predictive modeling of the outcomes and efficiency of edits, including off-target activity (reviewed by Pacesa et al., 2024). Applications of AI tools has huge potential in future for developing more efficient gene editing approaches to overcome current challenges discussed already and crucial to avoid unintended genetic alterations, particularly for therapeutic applications in various disease, including CNS disorders like AD, where safety and efficacy are of paramount interest. Although, the potential applications of AI in gene editing are immense but adoption will require careful consideration moving forward considering its limitations.

Regarding problems of efficient delivery systems for CRISPR/Cas9 in AD, use of nanoparticles-based carriers for CRISPR/Cas9 to specific body parts or cells have been reported and are mentioned elsewhere under section 2.2 of this review. Gold nanoparticles provide a perfect option for drug administration owing to their special attributes, which include small size, stability, and biocompatibility. The ability to readily functionalize gold nanoparticles to target particular cells or tissues is one of the benefits of employing them for drug delivery. Targeted delivery of CRISPR/Cas9 can be facilitated by affixing ligands for cell surface receptors to the nanoparticles surface. A fascinating area of research in the realm of nanobiotechnology is the coupling of gold nanourchins and CRISPR/Cas9. Gold nanourchins are nanoscale formations of gold atoms with a distinctive urchin-like shape. They provide an ideal platform for applications, such as medication delivery, imaging, and sensing, owing to their enormous surface area and ability to be functionalized with a variety of biomolecules. The encapsulation of CRISPR/Cas9 within gold nanourchins might significantly enhance CRISPR/Cas9 delivery to cells and tissues besides preventing degradation, and stands a good chance for enabling gene-editing treatments. Despite the tremendous promise for therapeutic approaches using gold nanoparticles or gold nanourchins, more research is needed to overcome several challenges before these nanocarriers can be effectively used. The possibility of their toxicity is a significant obstacle considering gold nanoparticles have the ability to accumulate in certain tissues and inflict damage. To ensure optimal efficacy and safety, their size, shape, and functionalization methods need to be optimized. The combination of gold nanoparticles and CRISPR/Cas9 holds immense potential in future medicine. However, further research is needed to fully evaluate the safety and effectiveness of such strategies with the ultimate goal of creating novel and efficient treatments for patients with a variety of illnesses (Lee et al., 2017 Joshi et al., 2022) and can be extended to CNS disorders including AD.

Another interesting approach in this regard was reported recently as advances in PBN delivery systems for CRISPR/Cas9, using a new generation of cell penetrating “ADGN peptides” that are fascinating and hold great promising (Gonzalez et al., 2024). ADGN nanoparticles were able to overcome delivery barrier, deliver a functional and efficient CRISPR Cas9 system having ability for systemic extrahepatic delivery of therapeutic nucleotides (Wickline et al., 2023). They offer chemical diversity and functional potential, compared to LNPs and thereby provide flexible structural designs to deliver different nucleic acid types as well as to develop targeting strategies. The successful delivery of CRISPR-Cas9 therefore demonstrated a great potential for the future of gene editing for clinical applications, which can be further optimized for effective Cas9 delivery *in vivo* for CNS disorders like AD.

## 14 Summary and conclusion

**FIGURE 5:**
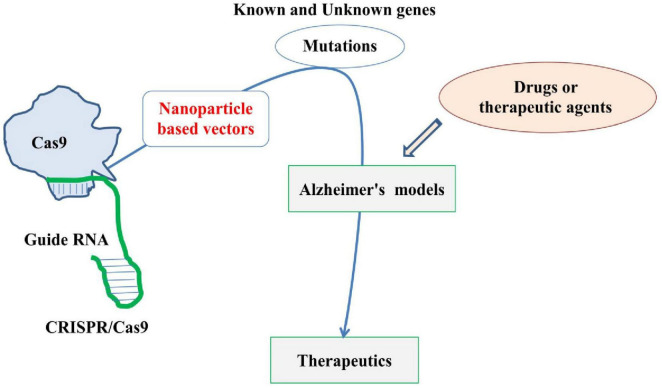
Basic and simplified diagram highlighting the application of CRISPR/Cas9 in developing Alzheimer’s disease (AD) model by carrying out various mutations and its therapeutic role. The figure also highlights the need for thoroughly investigating nanoparticles-based vectors (red) for CRISPR/Cas9.

The CRISPR/Cas9 technology has been transforming the field of CNS disease research, enabling unprecedented precision in editing genes and uncovering novel therapeutic targets. From neurodegenerative diseases to psychiatric disorders, CRISPR/Cas9 provides a versatile tool for modeling diseases, conducting high-throughput genetic screens, and validating potential drug targets. While therapeutic applications of CRISPR/Cas9 for CNS diseases are still in early stages, the technology holds immense promise for future treatments aimed at correcting genetic defects and modulating disease pathways. Using this technology, mutations in various known biomarkers, especially genes, are carried out to create suitable genetic models of AD, which are being used in assessing various aspects of AD, including the effects of various novel drugs and therapeutic agents. Various mutations can be introduced in AD models generated using the CRISPR/Cas9 technique to study their therapeutic impact (Figure 5). For example, mutation of the *APP* gene at specific regions to create an AD model followed by induction of other mutations or reversal of the original mutation can help in better understanding of the roles of such genetic markers. Further, to overcome challenges of efficient delivery mechanism for CRISPR/Cas9, this system can be combined with nanotechnology based carriers, especially the gold particle-based nano carriers and highly penetrating peptide nanoparticles for enhanced delivery, therapeutic efficacy and safety. With continued research and development, this strategy holds great promise in revolutionizing the field of gene therapy and precision medicine in CNS disorders like AD.
